# The impact of knowledge of β‐amyloid PET scan result on cognitive test performance: results of the REVEAL‐SCAN study

**DOI:** 10.1002/alz.089271

**Published:** 2025-01-03

**Authors:** Jason Karlawish, Kristin Harkins, Subhamoy Pal, Kathleen A. Welsh‐Bohmer, J. Scott Roberts, Allyson Gregoire, Jonathan M Reader, Kelly M. Bakulski, Robert C. Green

**Affiliations:** ^1^ University of Pennsylvania, Philadelphia, PA USA; ^2^ Michigan Alzheimer’s Disease Research Center, Ann Arbor, MI USA; ^3^ Duke University Medical Center, Durham, NC USA; ^4^ University of Michigan School of Public Health, Ann Arbor, MI USA; ^5^ Brigham and Women’s Hospital, Boston, MA USA

## Abstract

**Background:**

The impact of knowledge of β‐amyloid status on cognitively unimpaired persons' cognitive test performance is unknown.

**Method:**

Cognitively unimpaired adults aged 65‐80 with a first‐degree relative with AD received a dementia risk estimate and were randomly assigned to disclosure (D+) or non‐disclosure (D‐) of their β‐amyloid PET scan result. At 6 weeks and 6 months post‐disclosure, participants completed the ADCS‐PACC, a composite of Free and Cued Selective Reminding (free portion), Logical Memory IIa test, Digit‐Symbol Substitution, and MMSE. Scores from each test are standardized to a Z‐score metric and a composite score calculated by summing the standardized Z‐scores.

**Result:**

315 participants were randomized (84 elevated (41 A+D+/43 A+D‐)) and 231 not elevated (118 A‐D+, 113 A‐D‐)). After adjusting for baseline PACC scores, analyses compared PACC scores between (1.) A+D+ and A+D‐ and (2.) A‐D+ and A‐D‐ and stratified by race. A+ participants who learned their result (D+) had lower mean follow‐up PACC scores than participants who did not learn their β‐amyloid PET scan result (D‐) at 6 weeks (β = ‐0.89, 95% CI: ‐1.65, ‐0.13, p = 0.023) but not at 6 months post‐disclosure (β = ‐0.50, 95% CI ‐1.28, 0.27, p = .21). Mean follow‐up PACC scores did not differ between A‐ who did or did not learn their result at both 6 weeks (β ‐0.02, 95% CI: ‐0.42, 0.37, p = 0.91) and 6 months post‐disclosure (β ‐0.15, 95% CI ‐0.54, 0.25, p = .47). Analyses stratified by race showed Black/African American A+D+ had lower scores than A+D‐ at 6 weeks (β ‐2.01, 95% CI: ‐3.57, ‐0.45, p = 0.01) and 6 months post‐disclosure (β ‐1.63, 95% CI ‐3.19, ‐0.06, p = .04). Disclosure had no impact on scores of nonblack participants with elevated amyloid and the scores of any of not elevated participant groups.

**Conclusion:**

Knowledge of an elevated amyloid result reduced cognitively unimpaired persons PACC scores. A greater impact was observed in Black/African American participants’ test performance compared to non‐Black participants. In contrast, knowledge of not‐elevated amyloid showed no impact on test performance in any of the groups. Clinical trials should consider the potential impact of AD biomarker knowledge on cognitive outcomes.

